# *Paracerella* Imadaté in China: the description of a new species and the analysis of genetic differences between populations (Protura, Acerentomata, Nipponentomidae)

**DOI:** 10.3897/zookeys.604.8737

**Published:** 2016-07-11

**Authors:** Yun Bu, Yao Ma, Yun-Xia Luan

**Affiliations:** 1 Natural History Research Center, Shanghai Natural History Museum, Shanghai Science & Technology Museum, Shanghai, 200041, China; 2 Key Laboratory of Insect Developmental and Evolutionary Biology, Institute of Plant Physiology and Ecology, Shanghai Institutes for Biological Sciences, Chinese Academy of Sciences, Shanghai, 200032 China; 3 University of Chinese Academy of Sciences, Beijing, China

**Keywords:** DNA barcodes, genetic divergences, identification key, sensillum, taxonomy

## Abstract

The genus *Paracerella* Imadaté, 1980 is recorded from China for the first time, with the description of a new species, *Paracerella sinensis* **sp. n.** *Paracerella sinensis* is characterized by four pairs of *A*-setae on tergite I, the presence of setae *Pc* and *P3a* on tergite VII, eight *A*-setae on tergite VIII, the presence of seta *Pc* on both sternites VI and VII, and 4/2 setae on sternite VIII, which are different from all other members of the genus. The key to the four species of the genus is updated. In addition, DNA barcodes of four populations are sequenced and their genetic differences are analyzed.

## Introduction

The genus *Paracerella* Imadaté, 1980 is separated from *Verrucoentomon* Rusek, 1974 by the parallel position of the foretarsal sensilla *d* and *a*’ to *t2*. It is characterized by having a distinct calyx with racemose appendices on its surface, three pairs of *A*-setae on meso- and metanota, filiform foretarsal sensillum *t1*, three *A*-setae on sternites I–VII, posterior position of setae *P3* on tergites II–VI, well-developed labial palps, two subequal setae on abdominal legs II and III and well-developed striate band on segment VIII.

As a small genus in Protura, *Paracerella* has only three known species: *Paracerella shiratki* (Imadaté, 1964) recorded from Japan (Imadaté 1964, 1980), *Paracerella americana* Imadaté, 1980, and *Paracerella monterey* Shrubovych, 2012 from USA (Imadaté 1980; Shrubovych and Smykla 2012).

During field work in Inner Mongolia and Heilongjiang Provinces, northeast China, plenty of proturan specimens of *Paracerella* were found. They were identified as a new species and are described in the present paper, and an updated key to the genus was also provided. In addition, the DNA barcodes of the new species from four localities were sequenced and analyzed, the morphological identification was confirmed, and the genetic differences between different populations were revealed.

## Materials and methods

Specimens were collected by Tullgren funnels. All specimens were mounted on slides in Hoyer’s medium and dried at 60 °C. Specimens were identified and drawn with the aid of a NIKON E600 phase contrast microscope. The photos were taken by digital camera Nikon DXM1200. Type specimens are deposited in the Shanghai Entomological Museum (SEM), Institute of Plant Physiology & Ecology, Shanghai Institutes for Biological Sciences, Chinese Academy of Sciences, and Shanghai Natural History Museum (SNHM).

Abbreviations used in the text follow the paper of Bu and Yin (2007). Head setae and pores are marked according to Rusek et al. (2012). Body setae are marked following Imadaté (1974) and Yin (1999). Terminology of body porotaxy follows Szeptycki (1988) and Shrubovych (2014). Arrangements of the taxa follow the system proposed by Yin (1999).

For the analysis of genetic differences, genomic DNA was extracted from each individual separately by means of a non-destructive method (Gao and Bu 2014). After the DNA extraction, the cuticles of proturans were retrieved and mounted on the slides as voucher specimens. DNA barcoding sequences of mitochondrial COI gene were amplified and sequenced by primer pair LCO/HCO (Folmer et al. 1994). The barcoding sequences are deposited in GenBank. The nucleotide composition and the genetic divergence based on the Kimura-2-parameter (K2P) model were calculated using MEGA 6 (Tamura et al. 2013).

## Taxonomy

### Paracerella sinensis sp. n.

Taxon classificationAnimaliaProturaNipponentomidae

http://zoobank.org/632E7390-7077-4E5E-813E-F4105969B390

**Figure 1.**
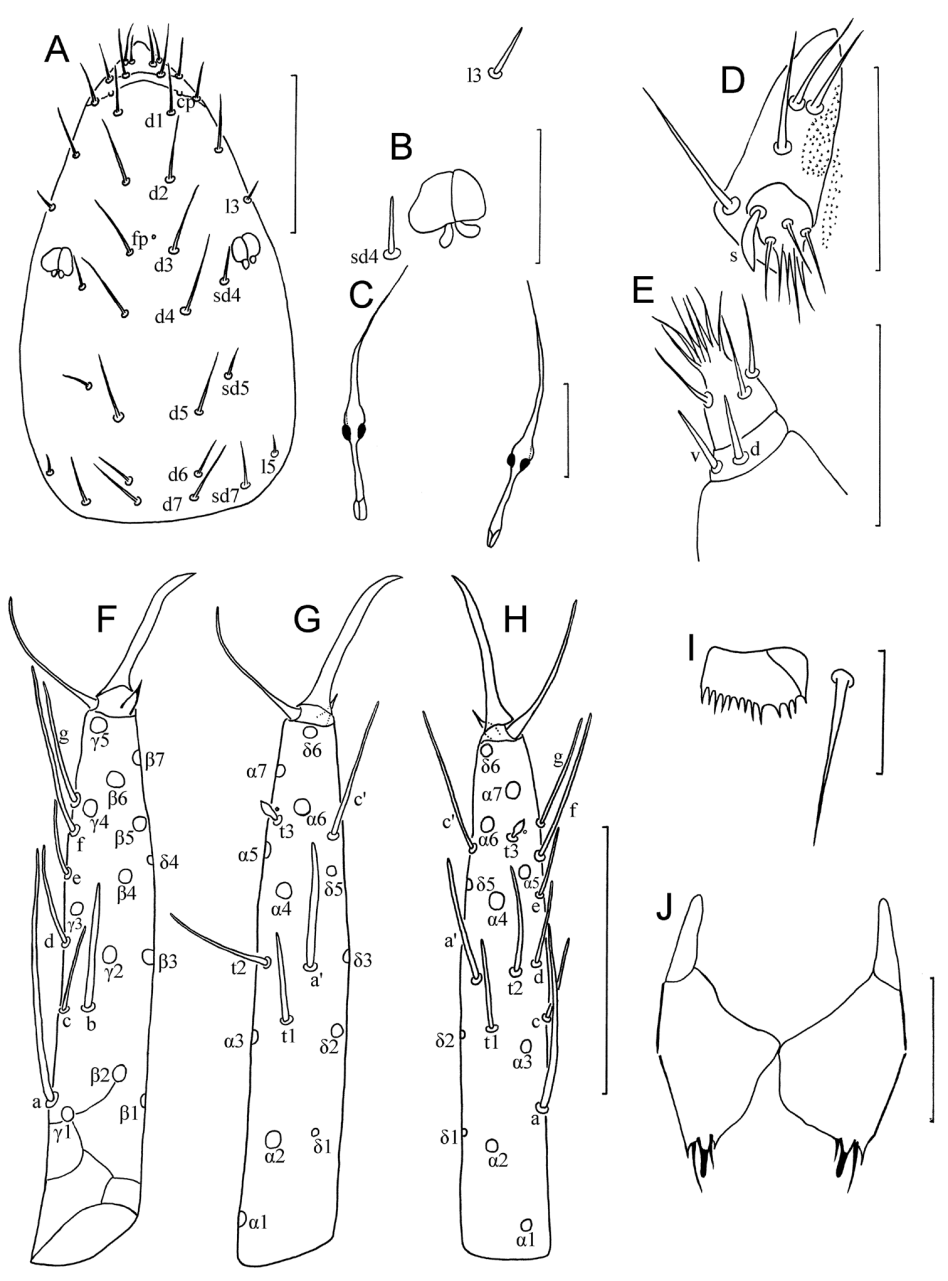
*Paracerella sinensis* sp. n. holotype. **A** Head, dorsal view (*cp* = clypeal pore, *fp* = frontal pore) **B** pseudoculus **C** canal of maxillary gland **D** labial palpus **E** maxillary palpus (d = dorsal sensillum, v = ventral sensillum) **F** foretarsus, exterior view **G** foretarsus, interior view **H** foretarsus, interolateral view (paratype No. LM6-14D) **I** comb **J** female quama genitalis. Scale bars: (**A, F–H**) 50 μm; others, 20 μm.

**Figure 2.**
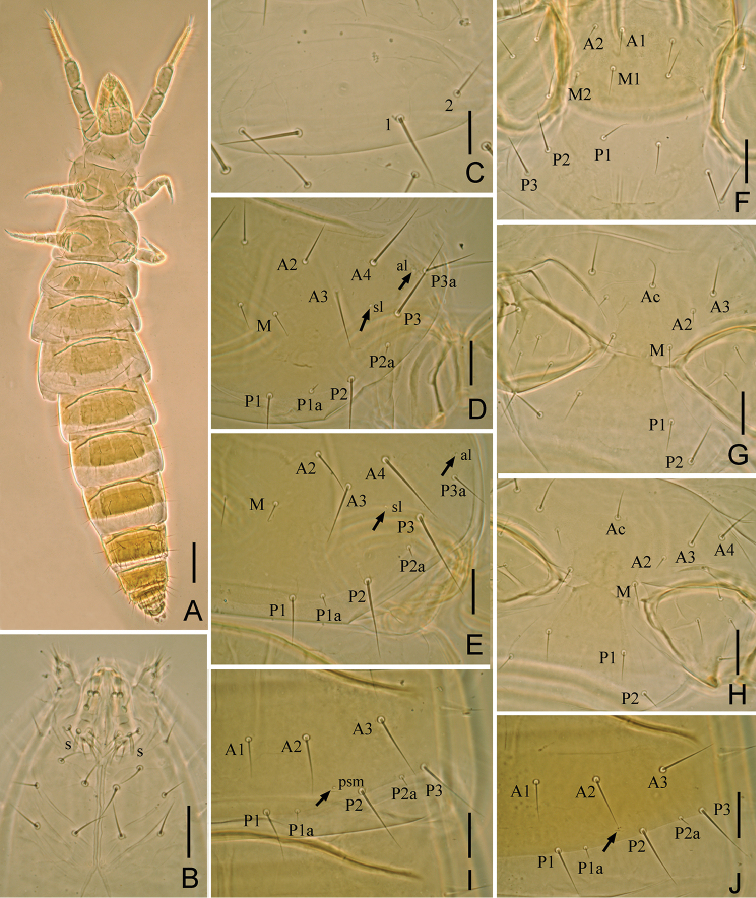
*Paracerella sinensis* sp. n. holotype. **A** Habitus **B** ventral side of head (s=sensillum) **C** pronotum **D** mesonotum **E** metanotum **F** prosternum **G** mesosternum **H** metasternum **I** tergite I, right side **J** tergite III, right side. Arrows indicate pores. Scale bars: (**A**)100 μm, others, 20 μm.

**Figure 3.**
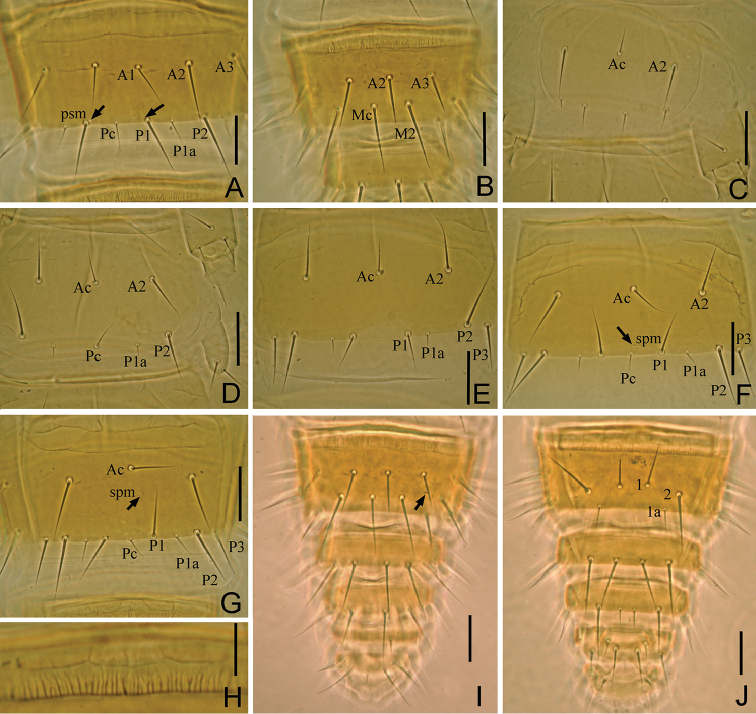
*Paracerella sinensis* sp. n. holotype. **A** Tergite VII (*psm*= posterosubmedial) **B** tergite VIII **C** sternite I **D** sternite II **E** sternite IV **F** sternite VI (s*pm*= sternal posteromedial) **G** sternite VII **H** striate band of abdominal VIII **I** Tergite VIII–XII **J** sternites VIII–XII. Arrows indicate pores. Scale bars: 20 μm.

Figs 1 , 2 , 3 , Tables 1 , 2

#### Material examined.

Holotype, female (No. LM6-12D) (SEM), **China**, Inner Mongolia Province, Balin town, Lama Hill, extracted from the soil samples under some small pine trees, 48°19.969'N, 122°19.160'E, elev. 562 m, 12-VIII-2014, coll. W.J. Chen, C.W. Huang, Y. Ma, Y.X. Luan, and M. Potapov. Paratypes, 4 females (nos. LM6-10, LM6-11, LM6-13D, LM6-14D) (SEM), same data as holotype; 3 females (nos. HH1-1D, HH1-3D, HH1-4D) (SEM), **CHINA**, Heilongjiang Province, Heihe City, from the soil samples under some black birches of Tree Farm 727, 50°15.491'N, 126°48.434'E, elev. 410 m, 15-VIII-2014; 11 females (nos. WHS4-2D, WHS5-2D, WHS6-2D, WHS4-6-1, WHS4-6-2, WHS5-3-2, WHS5-4-1, WHS5-4-3 in SEM, nos. WHS4-5-1, WHS4-5-2, WHS4-5-3 in SNHM), **China**, Heilongjiang Province, Wudalianchi City, from three soil samples of Wohu Hill, 48°39.252'N, 126°02.281'E, elev. 480 m, 17-VIII-2014; 5 females (nos. DZH2-1D, DZH2-2D, DZH2-3, DZH2-12D, DZH2-16) (SEM), **China**, Heilongjiang Province, Wudalianchi City, from the soil sample under some larches in Dazhanhe National Forest Park, 48°41.726'N, 127°40.556'E, elev. 327 m, 18-VIII-2014. Other materials,1 maturus junior (no. HH7-1) (SEM), **China**, Heilongjiang Province, Heihe City, from the soil samples under some black oaks of Tree Farm 733, 50°13.909'N, 126°51.887'E, elev. 517 m, 15-VIII-2014; 1 maturus junior (no. WHS6-3-2) (SEM), **China**, Heilongjiang Province, Wudalianchi City, from three soil samples of Wohu Hill, 48°39.252'N, 126°02.281'E, elev. 480 m, 17-VIII-2014; 3 maturi juniores (nos. DZH2-18, DZH 2-19, DZH2- 20) (SEM), 2 larvae II (nos. DZH2-4, DZH2-17) (SEM), **China**, Heilongjiang Province, Wudalianchi City, from the soil sample under some larches in Dazhanhe National Forest Park, 48°41.726'N, 127°40.556'E, elev. 327 m, 18-VIII-2014. All specimens are collected by W. J. Chen, C.W. Huang, Y. Ma, Y.X. Luan, and M. Potapov. Twelve specimens (nos. LM6-12D, LM6-13D, LM6-14D, HH1-1D, HH1-3D, HH1-4D, WHS4-2D, WHS5-2D, WHS6-2D, DZH2-1D, DZH2-2D and DZH2-12D) are voucher specimens retrieved after DNA extraction.

#### Diagnosis.

*Paracerella sinensis* sp. n. is characterized by four pairs of *A*-setae on tergite I, the presence of seta *Pc* and *P3a* on tergite VII, 8 *A*-setae on tergite VIII, the presence of seta *Pc* on sternites VI and VII, 4/2 setae on sternite VIII, which are different to any other members of the genus, foretarsal sensillum *a* extremely long, surpassing base of sensillum *e*, sensilla *d* and *a*’ located in subequal level with *t2*, acrostyli of female squama genitalis each with two fine flaps.

#### Description.

Adult body length 1150–1450 μm (n = 24), body yellow-brown color (Fig. 2A).

*Head* (Fig. 1A). Ovate, length 140–150 μm, width 85–90 μm. Setae *d6* present, *sd4* and *sd5* short, sensilliform. Setae *d6* 14–15 μm, *d7* 17–18 μm. Clypeal pore *cp* and frontal pore *fp* present. Pseudoculus round, length 8–10 μm, with short posterior extension, PR = 15–19 (Fig. 1B). Maxillary gland large, calyx with lateral racemose appendices and one helmet-like dorsal appendix, and bilobed posterior dilation, posterior filament length 15–17 μm, CF = 8–10 (Fig. 1C). Labial palpus well-developed, with tuft and one leaf-shaped basal sensillum (7–8 μm) (Figs 1D, 2B). Maxillary palpus with two tapering sensilla, subequal in length (8–9 μm) (Fig. 1E).

*Foretarsus* (Fig. 1F, G, H). Length 100–107 μm, claw length 20–25 μm, TR = 4.2–5.2; empodium length 5 μm, EU = 0.2–0.25. Dorsal sensilla *t1* filiform, BS = 0.75–0.85, *t2* slender and long (25 μm), *t3* lanceolate and short. Exterior sensilla *a* broad and long (35–55 μm), surpassing base of *d*, *b* broader and longer than *c*, *c* short and slender, *d* filiform, same level to *t2* and *a*’, *e* short, *f* and *g* filiform and long. Interior sensilla *a*’ broad and long, surpassing base of *δ5*, *b*’ absent, *c*’ slender and surpassing base of claw. Relative length of sensilla: *t3* < *e < c < d < t1<* (*b = g = t2*)< *a*’ < *c*’ < *f* < *a*. Setae *β1* (9 μm) and *δ4* (13 μm) setiform. Pores close to sensilla *c* and *t3* present. Length of middle tarsus 50 μm, claw length 23–25 μm. Length of hind tarsus 55 μm, claw length 25 μm.

*Thorax*. Thoracic chaetotaxy given in Table 1. Setae *1* and *2* on pronotum 31 μm and 21 μm length respectively (Fig. 2C). Mesonotum and metanotum with eight pairs of *P*-setae, accessory setae short sensilliform, 6–8 μm in length; setae *P1*, *P1a* and *P2* on mesonotum 21–25 μm, 5–6 μm and 35–36 μm respectively (Fig. 2D, E). Prosternum with two pairs of anterior seta, and setae *A2* and *M2* sensilliform, 6–7 μm (Fig. 2F). Mesosternum and metasternum with 5 and 7 *A*-setae respecitvely, and setae *A2* on mesosternum and metasternum sensilliform, 6–7 μm (Fig. 2G, H). Pronotum without pores. Mesonotum and metanotum with pores *sl* and *al*. Sterna without pores.

**Table 1.** Adult chaetotaxy of *Paracerella sinensis* sp. n.

Segment		Dorsal		Ventral	
		Formula	Setae	Formula	Setae
Th.	I	4	1, 2	4 + 4 6	A1, 2, M1, 2 P1, 2, 3
	II	8 16	A2, 3, 4, M P1, 1a, 2, 2a, 3, 3a, 4, 5	5 + 2 4	Ac, 2, 3, M P1, 2
	III	8 16	A2, 3, 4, M P1, 1a, 2, 2a, 3, 3a, 4, 5	7 + 2 4	Ac, 1, 2, 3, M P1, 2
Abd.	I	8 12	A1, 2, 3, 5 P1, 1a, 2, 2a, 3, 4	3 4	Ac, 2 P1, 1a
	II–III	10 16	A1, 2, 3, 4, 5 P1, 1a, 2, 2a, 3, 4, 4a, 5	3 5	Ac, 2 Pc, 1a, 2
	IV–V	10 16	A1, 2, 3, 4, 5 P1, 1a, 2, 2a, 3, 4, 4a, 5	3 8	Ac, 2 P1, 1a, 2, 3
	VI	10 16	A1, 2, 3, 4, 5 P1, 1a, 2, 2a, 3, 4, 4a, 5	3 9	Ac, 2 Pc, 1, 1a, 2, 3
	VII	10 19	A1, 2, 3, 4, 5 Pc,1, 1a, 2, 2a, 3,3a, 4, 4a, 5	3 9	Ac, 2 Pc, 1, 1a, 2, 3
	VIII	8 15	A1, 2, 4, 5 Mc, 2, 3, 4, P2, 3, 4, 5	4 2	1, 2 1a
	IX	12	1, 1a, 2, 2a, 3, 4	4	1, 2
	X	10	1, 2, 2a, 3, 4	4	1, 2

*Abdomen*. Abdominal chaetotaxy given in Table 1. Tergite I with four pairs of anterior setae (*A1*, *A2*, *A3*, *A5*) (Fig. 2I). Tergites II–VI with five pairs of anterior setae and eight pairs of posterior setae (Fig. 2J). Tergite VII with five pairs of anterior setae and 19 posterior setae, *Pc* and *P3a* present (Fig. 3A). Accessory setae on tergites I–VI short sensilliform, 6–7 μm on tergites I–III, 7–9 μm on tergite IV–VI, and on VII setiform (13–16 μm). Tergite VIII with seta *Mc* (Fig. 3B). Sternites IV–V each with eight posterior setae (Fig. 3E). Sternites VI–VII each with nine posterior setae, *Pc* present (Fig. 3F, G). Sternite VIII with two rows of setae (4/2) (Fig. 3J). Hind margin of tergites IX–XI and sternites IX–X with distinct denticles.

Tergites I–III and VII with pores *psm* and *al*, IV–VI with pores *psm*, *al* and *psl*, VIII with pores *psm* only, IX–XI without pores, XII with single medial pore. Pores *psm* on tergite VII close to seta *P1* (Fig. 3A). Sternites I–V without pores (Fig. 3C, D, E), VI and VII each with single medial pore *spm*, on VI located close to *Pc* and on VII located in central position (Fig. 3F, G). Sternites VIII–XI without pores, XII with 1+1 *sal* pores.

Abdominal appendages I, II, III with 2, 1, 1 segments and 4, 2, 2 setae respectively (Fig. 3C, D). On appendages II and III, subapical setae 19–21 μm, apical setae 18–19 μm in length. Striate band on abdominal segment VIII well-developed (Fig. 3H, I, J). Comb on abdomen VIII rectangular, with 12–13 teeth (Fig. 1I). Female squama genitalis robust, with short basal apodeme and pointed acrostyli, each acrostylus with two fine flaps (Fig. 1J). Male unknown.

#### Etymology.

The species is named after the Latin name of China, the place where the species was found.

#### Distribution.

Inner Mongolia and Heilongjiang, China.

#### Remarks.

The new species is placed in the genus *Paracerella* because of the three pairs of *A*-setae on both meso- and metanota, filiform sensillum *t1* on foretarsus, sensilla *d* and *a*’ located in subequal level with *t2*, and well-developed striate band. *Paracerella sinensis* sp. n. can be easily distinguished from the other three species of the genus by the chaetotaxy of tergites I, IV and VIII, sternites VI–VIII, as well as the length of foretarsal sensillum *a*.

Among 24 adults of *Paracerella sinensis* observed, the length of sensillum *a* is variable between individuals: in most specimens it can surpass base of *e* (holotype and most of paratypes) (Fig. 1F), in some specimens it is a little shorter, only surpassing base of *d* (nos. LM6-10, LM6-14D) (Fig. 1H), in some specimens it is extremely long as reaching or surpassing base of *f* (nos. LM6-13D, HH2-4D, WHS4-6-1), even reaching base of *g* (no. WHS4-2D). The four species of *Paracerella* can be distinguished by the following key.

### Key to the species of genus *Paracerella* Imadaté, 1980

**Table:** 

1	Tergites I–VI with seta *P1a*, sternite I with 4 posterior setae	**2**
–	Tergites I–VI without seta *P1a*, sternite I with 2 posterior setae	***Paracerella monterey* Shrubovych, 2012**; USA (California)
2	Tergite VII without *Pc* and *P3a* setae, sternite VI without *Pc* seta	**3**
–	Tergite VII with *Pc* and *P3a* setae, sternite VI with *Pc* seta	***Paracerella sinensis* sp. n.**; China (Inner Mongolia, Heilongjiang)
3	Tergite VII with 8 *A*-setae and without seta *P1a*, tergite VIII with 6 *A*-setae	***Paracerella americana* Imadaté, 1980**; USA (California)
–	Tergite VII with 6 *A*-setae and with *P1a* seta, tergite VIII with 4 *A*-setae	***Paracerella shiratki* (Imadaté, 1964)**; Japan (Hokkaido)

### Genetic differences between populations of *Paracerella sinensis* sp. n.

The standard DNA barcoding sequences (COI genes) of eight individuals (voucher species nos. LM6-12D, LM6-14D, HH1-2D, WHS4-2D, WHS5-2D, WHS6-2D, DZH2-1D and DZH2-2D) from one locations in Inner Mongolia (LM) and three locations in Heilongjiang (HH, WHS and DZH) were sequenced and deposited in GenBank (accession numbers KU983757-KU983764). Each sequence contains 658 base pairs of nucleotides that encoding 219 amino acids. The average nucleotide composition is A = 25.2%, T = 41.5%, C = 15.9%, and G = 17.4%.

The K2P genetic divergences of nucleotides for barcode sequences are 0-3.78% between individuals within the same population, and 0.46%-12.54% between individuals from different populations. The numbers of different coded amino acids for this sequence are 0-3 between individuals within the same populations, and 1-4 between individuals from different populations. Except that the COI gene sequence of WHS4-2D is more similar to COI of HH1-2D than to COI of WHS5-2D and WHS6-2D, our data show low genetic variation within populations (LM, WHS, and DZH), but reveal high genetic differentiation among four geographic populations (Table 2).

**Table 2.** The K2P genetic distances of DNA barcodes (COI gene) in *Paracerella sinensis* sp. n.

	LM6-12D	LM6-14D	HH1-2D	WHS4-2D	WHS5-2D	WHS6-2D	DZH2-1D	DZH2-2D
LM6-12D								
LM6-14D	0.0000							
HH1-2D	0.1211	0.1211						
WHS4-2D	0.1173	0.1173	0.0046					
WHS5-2D	0.1251	0.1251	0.0346	0.0362				
WHS6-2D	0.1193	0.1193	0.0362	0.0378	0.0046			
DZH2-1D	0.1235	0.1235	0.1214	0.1214	0.1197	0.1178		
DZH2-2D	0.1254	0.1254	0.1233	0.1233	0.1216	0.1197	0.0015	

Note: The geographic distances among four populations are 397 km, 390 km, 277 km, 187 km, 185 km, and 121 km for LM-DZH, LM-HH, LM-WHS, HH-WHS, HH-DZH and WHS-DZH, respectively.

## Discussion

The intraspecific distances of most insects are very low. Virgilio et al. (2010) studied the 15,948 DNA barcodes involving 1,995 insect species across six insect orders (Coleoptera, Diptera, Hemiptera, Hymenoptera, Lepidoptera and Orthoptera), and found 95% of all intraspecific K2P distances ranging from 0 to 7.64%. However, the intraspecific genetic distances of *Paracerella sinensis* sp. n. are very high (up to 12.54%), which is in accord with the previous studies on some other proturan species: up to 21.3% in eight individuals of *Ionescuellum haybachae* from two Austria populations (Resch et al. 2014), and up to 31.98% separating 21 representatives of *Acerentomon italicum* in three Italian populations from an Austrian population (Galli et al. 2015). The similar situation was also found in another basal hexapod group–Collembola: six collembolan species sampled from various locations worldwide with high intraspecific variation for COI from 11.33% to 21.47% (Porco et al. 2012). Compared with insects, basal hexapods are more ancient, and probably accumulated more random genetic mutations. Another possible reason is the lack of gene flow due to the low dispersal ability of basal hexapods. Anyway, we need more data to compare the difference between intra- and interspecific divergence, for the evaluation of the standard DNA barcoding efficacy in Protura.

## Supplementary Material

XML Treatment for Paracerella sinensis
